# Design of Na_3_MnZr(PO_4_)_3_/Carbon Nanofiber Free-Standing Cathodes for Sodium-Ion Batteries with Enhanced Electrochemical Performances through Different Electrospinning Approaches

**DOI:** 10.3390/molecules29081885

**Published:** 2024-04-20

**Authors:** Debora Maria Conti, Claudia Urru, Giovanna Bruni, Pietro Galinetto, Benedetta Albini, Chiara Milanese, Silvia Pisani, Vittorio Berbenni, Doretta Capsoni

**Affiliations:** 1 Department of Chemistry, Physical Chemistry Section & C.S.G.I. (Consorzio Interuniversitario per lo Sviluppo dei Sistemi a Grande Interfase), University of Pavia, Via Taramelli 16, 27100 Pavia, Italy; deboramaria.conti01@universitadipavia.it (D.M.C.); claudia.urru01@universitadipavia.it (C.U.); giovanna.bruni@unipv.it (G.B.); chiara.milanese@unipv.it (C.M.); vittorio.berbenni@unipv.it (V.B.); 2 Department of Physics, University of Pavia, 27100 Pavia, Italy; pietro.galinetto@unipv.it (P.G.); benedetta.albini@unipv.it (B.A.); 3 Department of Drug Sciences, University of Pavia, Via Taramelli 12, 27100 Pavia, Italy; silvia.pisani@unipv.it

**Keywords:** Na_3_MnZr(PO_4_)_3_, cathode, sodium-ion batteries, electrospinning, carbon nanofibers, free-standing

## Abstract

The NASICON-structured Na_3_MnZr(PO_4_)_3_ compound is a promising high-voltage cathode material for sodium-ion batteries (SIBs). In this study, an easy and scalable electrospinning approach was used to synthesize self-standing cathodes based on Na_3_MnZr(PO_4_)_3_ loaded into carbon nanofibers (CNFs). Different strategies were applied to load the active material. All the employed characterization techniques (X-ray powder diffraction (XRPD), scanning electron microscopy (SEM), transmission electron microscopy (TEM), energy-dispersive X-ray spectroscopy (EDS), thermal gravimetric analysis (TGA), and Raman spectroscopy) confirmed the successful loading. Compared to an appositely prepared tape-cast electrode, Na_3_MnZr(PO_4_)_3_/CNF self-standing cathodes demonstrated an enhanced specific capacity, especially at high C-rates, thanks to the porous conducive carbon nanofiber matrix. Among the strategies applied to load Na_3_MnZr(PO_4_)_3_ into the CNFs, the electrospinning (vertical setting) of the polymeric solution containing pre-synthesized Na_3_MnZr(PO_4_)_3_ powders resulted effective in obtaining the quantitative loading of the active material and a homogeneous distribution through the sheet thickness. Notably, Na_3_MnZr(PO_4_)_3_ aggregates connected to the CNFs, covered their surface, and were also embedded, as demonstrated by TEM and EDS. Compared to the self-standing cathodes prepared with the horizontal setting or dip–drop coating methods, the vertical binder-free electrode exhibited the highest capacity values of 78.2, 55.7, 38.8, 22.2, 16.2, 12.8, 10.3, 9.0, and 8.5 mAh/g at C-rates of 0.05C, 0.1C, 0.2C, 0.5C, 1C, 2C, 5C, 10C, and 20C, respectively, with complete capacity retention at the end of the measurements. It also exhibited a good cycling life, compared to its tape-cast counterpart: it displayed higher capacity retention at 0.2C and 1C, and, after cycling 1000 cycles at 1C, it could be further cycled at 5C, 10C, and 20C.

## 1. Introduction

In a world rapidly moving towards renewable energy sources for limiting global climate change and promoting the transition to less carbon-intensive and more sustainable energy systems, the development of efficient energy storage devices to integrate intermittent energy production is a key issue [1,2]. Undoubtedly, rechargeable batteries have been regarded as promising energy storage systems, and, among them, lithium-ion batteries (LIBs) occupy a pivotal position, thanks to their high energy density, operating voltage, specific capacity, and long cycle life [3,4,5,6,7]. However, the non-ubiquitous distribution of lithium resources and their depletion inevitably cause price increases and pose concerns for large-scale applications [8,9,10,11,12,13]: for these reasons, alternative energy storage systems involving less critical elements need to be explored. Sodium-ion batteries (SIBs) seem to be a valuable choice, as sodium is a ubiquitous, abundant, and low-cost element [14,15]. Notably, sodium and lithium present comparable chemical and physical properties and a similar intercalation chemistry. Despite these appealing features and the progress recently achieved, several shortcomings still need to be addressed to develop SIBs with high energy and power densities, good lifespans, and fast sodium-ion diffusion and kinetics [16]. Among the battery components, cathodes play a key role, and the development of new and performant cathode materials is necessary to address the abovementioned drawbacks. Layered/tunneled transition metal oxides have been widely investigated as cathodes for SIBs, but the volume changes upon sodiation/desodiation and phase instabilities pose concerns about their application [17,18,19,20,21,22]. Prussian blue analogues are promising for their low cost, easy synthesis, and fast Na^+^ migration within the crystal network [23,24,25,26,27]. Polyanionic compounds were revealed to be suitable cathode materials, as they offer a robust three-dimensional polyanionic framework with tunnels for ion migration [28,29,30,31]. Among them, NASICON-structured cathodes have been widely investigated, thanks to the possibility to tune electrochemical performance by varying the transition metal ion. Compounds such as Na_3_V_2_(PO_4_)_3_, Na_3_Cr_2_(PO_4_)_3_, and Na_3_Fe_2_(PO_4_)_3_ were investigated and modified by carbon coating or doping to improve their low electronic conductivity [32,33,34,35]. Recently, research efforts have focused on NASICON-type mixed-transition metal phosphates, such as Na_3_Mn^2+^M(PO_4_)_3_ (M = tetravalent metal ion), in which high discharge voltages are achieved thanks to the reversible redox processes involving both Mn^4+^/Mn^3+^ and Mn^3+^/Mn^2+^ couples [36,37,38,39,40]. Among these phosphates, the Na_3_MnZr(PO_4_)_3_ cathode exhibits superior electrochemical performance [38,41]: it provides high voltage plateaus at 4.1 V and 3.5 V, corresponding to the reversible extraction of two Na^+^ ions with small changes in the cell volume. Na_3_MnZr(PO_4_)_3_ crystallizes in the rhombohedral $R\bar{3}c$ space group with lattice parameters *a* = 8.988 Å and *c* = 22.598 Å. Mn and Zr randomly occupy the octahedral sites of the NASICON structure. The Mn/Zr octahedra connect via oxygen to the PO_4_ tetrahedra to form a 3D network with three interstitial sites accessible for Na^+^ ions: Na(1), Na(2), and Na(3). These sites are partially occupied, and the Coulombic repulsion of the Na^+^ ions in Na(1) and Na(2) causes the displacement of Na^+^ ions from the Na(1) to Na(3) site. The sodiation and desodiation processes involve the Na(2) site. Gao and coworkers [38] reported a detailed investigation of the intercalation/deintercalation mechanism and demonstrated the suppression of the Jahn–Teller distortion of Mn^3+^ in Na_3_MnZr(PO_4_)_3_ compared to other Mn-based phosphates. Ma and coworkers [41] reported that the electrochemical performance of the Na_3_MnZr(PO_4_)_3_ cathode can be further improved by preparing Na_3_MnZr(PO_4_)_3_ microspheres with an embedded dual-carbon-based material (amorphous carbon and reduced graphene oxide).

In recent years, carbon nanofibers (CNFs) have been successfully employed in different areas such as energy [42], biomedicine [43,44], gas storage/separation [45,46], adsorption [47,48], and catalysis [49]. In the energy field, CNFs are well employed in LIBs and SIBs as anodes [50] or as the conducive filler and matrix of electrode materials [51,52,53,54,55,56]. CNFs are good electronic conductors and display a high surface area. They are easily synthesized by electrospinning and the obtained non-woven sheets possess a desirable porosity, favoring good electrolyte permeation and allowing the volume changes occurring during the sodiation/desodiation processes. Moreover, thanks to their good electronic conductivity and mechanical properties, CNFs are suitable for fabricating free-standing electrodes, avoiding the employment of Al foils as active material supports and current collectors. The active material can be loaded into CNFs by different approaches: (i) it is pre-synthesized and then dispersed in the polymeric solution to be electrospun, and, subsequently, the obtained sheet undergoes a carbonization process [54]; (ii) a solution containing the precursors of the active material is added to the polymeric solution to be electrospun, and the obtained sheets are thermally treated to carbonize, leading to the formation of the final product [51,52,53]; and (iii) the active materials’ precursor solution is added by a dip–drop method to pre-synthesized CNFs, and the soaked sheet is thermally treated to synthesize the active material into CNFs [55,56].

In this paper, we prepared and characterized, for the first time in the literature to our knowledge, Na_3_MnZr(PO_4_)_3_/CNF self-standing cathodes, and we tested their electrochemical performances for applications in SIBs. Na_3_MnZr(PO_4_)_3_ was chosen, as it is an appealing high-voltage cathode material compared to other mixed-transition metal phosphates [38,40,41]. CNFs are good electronic conductors; the electrolyte easily permeates their porous structure, and electrolyte–active material contact is facilitated. Based on the aforementioned CNF features, improved electrochemical performance with respect to conventional tape-cast cathodes is envisaged especially at high C-rates (≥10C), where the required fast ion diffusion is invalidated in packed materials [57,58]. The self-standing cathodes were prepared by electrospinning using both the dip–drop method on pre-electrospun CNFs and the dispersion of pre-synthesized Na_3_MnZr(PO_4_)_3_ in the polymeric solution to be electrospun. Different electrospinning settings were also applied (horizontal and vertical settings). The obtained composites were characterized by several techniques to investigate structure, morphology, composition, and Na_3_MnZr(PO_4_)_3_ distribution into the CNFs. The electrochemical performance of the free-standing electrodes was evaluated and compared to that of a conventional tape-cast cathode (70% active material), appositely prepared. We expected to obtain improved electrochemical performance for the self-standing cathodes, especially at high C-rates. Based on the physico-chemical features and electrochemical results, we could also identify the most efficient synthetic approach to prepare binder-free electrodes.

## 2. Results

### 2.1. p-MnZr and MnZr/CNF Characterization

**Figure 1:**
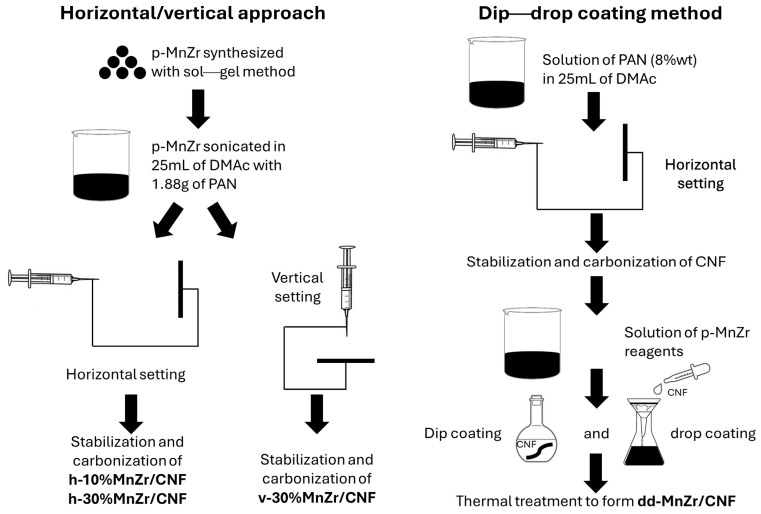
A scheme of the samples’ synthesis.

Pristine Na_3_MnZr(PO_4_)_3_ powder was synthesized via the sol–gel method [38] and used as the active material source in preparing both tape-cast electrode and some of the free-standing cathodes. Two different approaches were applied to prepare the self-standing electrodes, differing on the method employed to load the active material onto the CNFs: (i) dip–drop coating of the Na_3_MnZr(PO_4_)_3_ precursors onto electrospun CNFs sheets, followed by thermal treatments to synthesize the active material, or (ii) electrospinning the dispersion of pristine Na_3_MnZr(PO_4_)_3_ powder synthesized via sol–gel into PAN in a N,N-dimethylacetamide (DMAc) solution. In the latter case, two different Na_3_MnZr(PO_4_)_3_ amounts (10 and 30 wt%) and two electrospinning settings (horizontal and vertical) were investigated. Also, pure CNFs were prepared for comparison. A scheme of the samples’ synthesis is shown in Figure 1, and details are reported in Section 3.2. The prepared samples and their alphanumeric codes are listed in Table 1.

**Figure 2:**
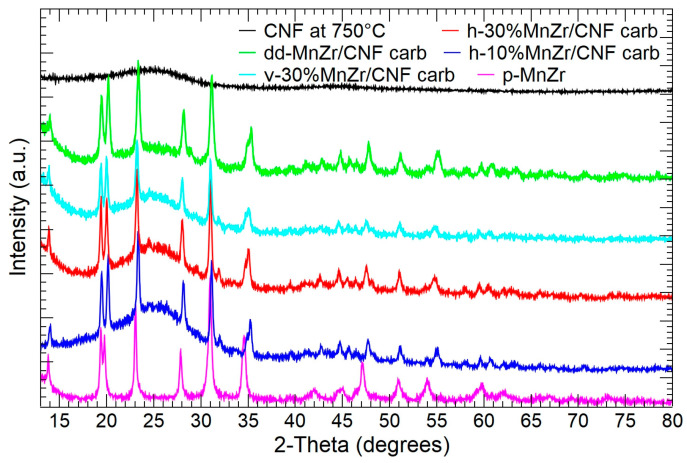
XRPD patterns of CNFs, pristine Na_3_MnZr(PO_4_)_3_ powder, and self-standing cathodes.

In Figure 2, the XRPD patterns of CNF, p-MnZr, dd-MnZr/CNF, h-10%MnZr/CNF, h-30%MnZr/CNF, and v-30%MnZr/CNF are shown. For the CNF sample, a broad band centered at a 2-theta value of about 25° is detected, as expected for amorphous components. The p-MnZr sample displays the peaks pertinent to the NASICON-type Na_3_MnZr(PO_4_)_3_ compound ($R\bar{3}c$ space group) deposited in the JCPDS database (PDF# 041-0504). The diffraction data are well explained by the literature structure given by Gao and co-workers [38], as demonstrated by the Rietveld refinement results reported in Table S1 and the graphical comparison in Figure S1. No extra peaks due to Mn and Zr long-range ordering are detected, and the random distribution of the transition metal ions on the octahedral site of the NASICON framework is confirmed [59,60]. For the MnZr/CNFs samples, both the broad band typical of the amorphous component and the reflections of the Na_3_MnZr(PO_4_)_3_ NASICON structure are observed, independently of the deposition method. The self-standing cathode obtained by the dip–drop approach is impurity-free, while those prepared by dispersing the active material into the PAN solution display a weak peak at 2-theta of about 32°: this signal is explained by a small amount of MnZr_4_(PO_4_)_6_ phase (PDF# 045-0016), possibly formed during the further thermal treatment at 750 °C for the carbonization process. The Rietveld refinement results for the MnZr/CNFs samples are reported in Table S1, and the graphical comparisons are shown in Figure S2. The crystalline component of the diffraction patterns is well explained by the Na_3_MnZr(PO_4_)_3_ phase. Notably, the *c* lattice parameter increases and *a* decreases compared to the pristine Na_3_MnZr(PO_4_)_3_ powder. This leads to a slight cell volume decrease and a *c*/*a* ratio increase (See Table S1). The crystallite size is slightly increased in the self-standing cathodes obtained by the dispersion approach, and it is consistent with the prolonged thermal treatment at 750 °C for the additional carbonization process, not required in the pristine and dip–drop synthesis routes.

**Figure 3:**
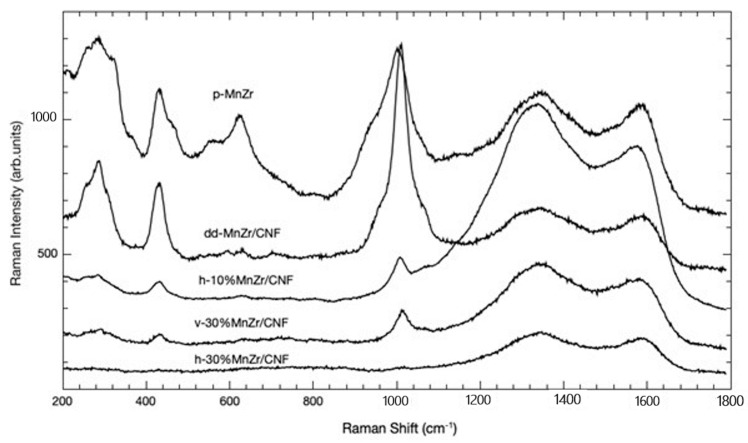
Raman spectra of p-MnZr, h-10%MnZr/CNF, h-30%MnZr/CNF, v-30%MnZr/CNF, and dd-MnZr/CNF samples.

To analyze the phase formation and composition of the composite samples, we also employed micro-Raman spectroscopy. The Raman spectra of the pristine Na_3_MnZr(PO_4_)_3_ and MnZr/CNFs samples are shown in Figure 3. In the case of highly diffusive powders, it is important to note that micro-Raman sampling is strongly effective in surface layers.

In all the cases, we can distinguish two different spectral regions: the first, in the range 200–1100 cm^−1^, presents Raman features ascribed to the active material [61], while the second, in the range 1100–1800 cm^−1^, presents the typical fingerprints of carbon-based structures, with the well-known D and G bands [62].

In the first spectral region, the typical spectrum of Na_3_MnZr(PO_4_)_3_ is observed for the pristine sample. The strong signal at around 1000 cm^−1^ is due to the overlapping of the Raman activity associated with PO_4_ symmetric (ν_1_) and asymmetric (ν_3_) stretching vibrations. At lower energies, different Raman activities can be present. The strong signal at around 430 cm^−1^ could be due to symmetric (ν_2_) bending vibrations of the PO_4_ unit, with the corresponding asymmetric (ν_4_) bending mode responsible for the lower-intensity signals around 540 cm^−1^. The bands observed in the 350−330 cm^−1^ range could be instead associated with metal–oxygen vibrations, while bands below 250 cm^−1^ are usually attributed to lattice vibrations. Even for the pristine sample, typical carbon-related Raman features are observed, possibly due to the carbon coating. 

Moreover, we can observe that the active material’s Raman fingerprint is clearly visible for the dd-MnZr/CNF sample, while it is attenuated for all the other ones. This is due to the different preparation methods leading to different in-depth profiles of the active material, as confirmed by the EDS analysis reported below. 

Concerning the carbon-related features, i.e., the bands at 1333 and 1586 cm^−1^, the former related to the disordered carbon, the latter to the G band, we observe an almost constant ratio between their intensities except for the h-10%MnZr/CNF sample, where the disorder, i.e., a greater I_D_/I_G_ ratio, is more pronounced.

**Figure 4:**
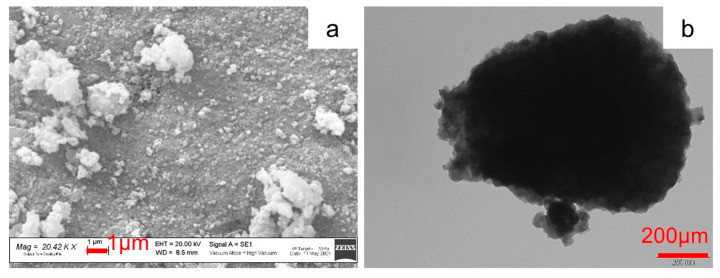
SEM and TEM images of Na_3_MnZr(PO_4_)_3_ powder: (**a**) 20.42 kX and (**b**) 100 kX.

Figure 4 shows the SEM and TEM images of the pristine Na_3_MnZr(PO_4_)_3_ powder. The SEM image shows large aggregates composed by nanometric sub-particles with a homogeneous morphology. The grains’ surface is irregular but defined. The TEM micrograph (Figure 4b) of the p-MnZr sample confirms the presence of aggregates of nanometric particles covered by a homogeneous carbon coating resulting from the citric acid decomposition occurred during sol–gel synthesis. 

**Figure 5:**
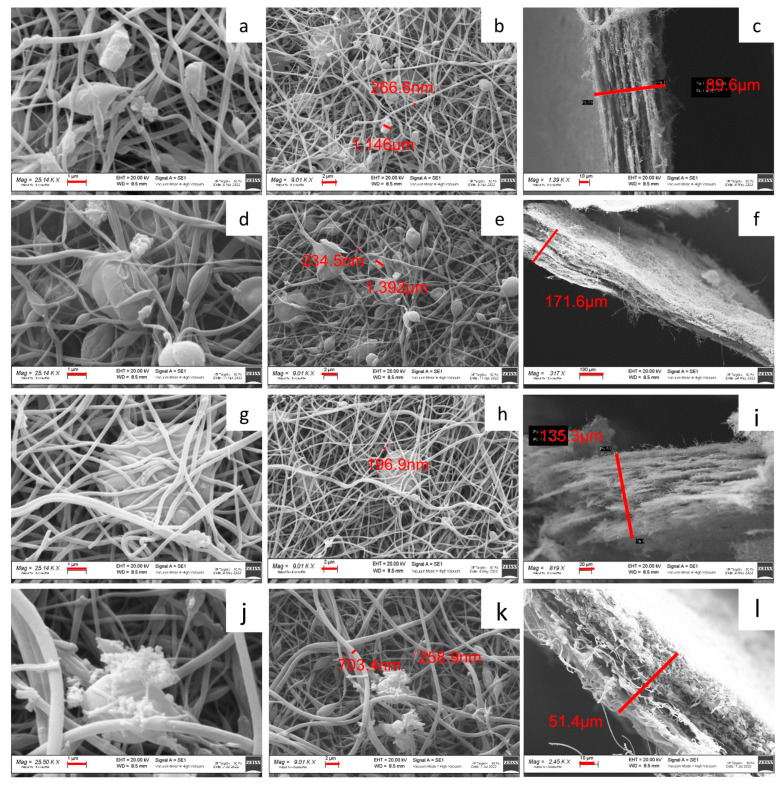
SEM images of h-10%MnZr/CNF (**a**,**b**) surface and (**c**) cross-section; h-30%MnZr/CNF (**d**,**e**) surface and (**f**) cross-section; v-30%MnZr/CNF (**g**,**h**) surface and (**i**) cross-section; and dd-MnZr/CNF (**j**,**k**) surface and (**l**) cross-section.

Figure 5 shows the SEM images of self-standing cathodes. For each sample, both the surface and the cross-section are investigated. All the self-standing electrodes are characterized by a matrix of non-woven carbon nanofibers, which hosts the Na_3_MnZr(PO_4_)_3_ aggregates of a micrometric size. Also, the carbon nanofibers display variable dimensions, averaging about 250 nm for the samples prepared dispersing the active material in the PAN solution before electrospinning and larger in the dip–drop sample (about 700 nm). The cross-section images put into evidence that a higher thickness (about 150 mm) is obtained for the 30 wt% samples, independently of the deposition setting. The sheets seem more compact and structured for the h-10%MnZr/CNF, h-30%MnZr/CNF, and v-30%MnZr/CNF samples and fluffier in the case of the dip–drop one. 

**Figure 6:**
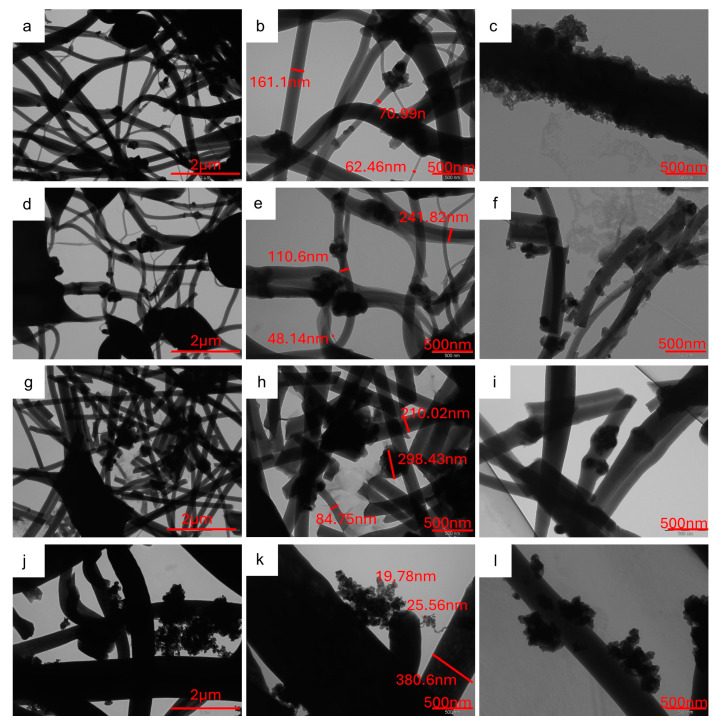
TEM images at different magnifications (20 kX e 50 kX) of (**a**–**c**) h-10%MnZr/CNF, (**d**–**f**) h-30%MnZr/CNF, (**g**–**i**) v-30%MnZr/CNF, and (**j**–**l**) dd-MnZr/CNF samples.

Figure 6 shows the TEM images of self-standing samples, which confirm the presence of active material agglomerates in CNFs, as evidenced by the SEM analysis. The aggregates are formed by sub-particles of about 20–40 nm, whose size compares to that evaluated by XRPD analysis (Table S1). Interestingly, in the samples prepared by the dispersion of pristine Na_3_MnZr(PO_4_)_3_ into the polymeric solution to be electrospun, the active material covers parts of the CNF surface but is also embedded into nanofibers; the latter feature is particularly evident in the v-30%MnZr/CNF sample. Differently, the agglomerates reside mainly on the CNFs surface and between them in the case of the dip–drop approach. This is consistent with the different synthetic route, in which the active material is formed in situ on carbonized electrospun nanofibers.

**Figure 7:**
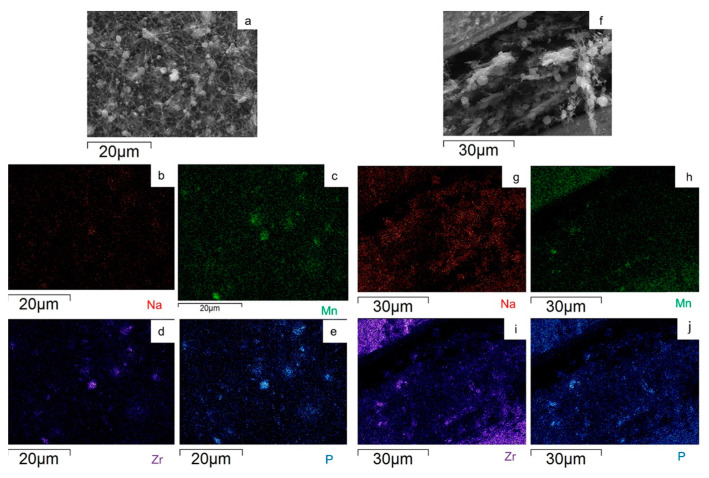
SEM image and EDS maps of the different elements for the h-10%MnZr/CNF (**a**–**e**) surface and (**f**–**j**) its cross-section.

**Figure 8:**
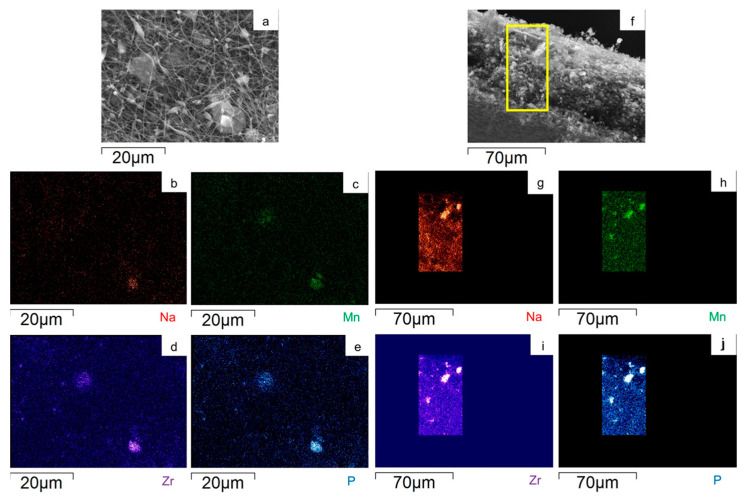
SEM image and EDS maps of the different elements for the h-30%MnZr/CNF (**a**–**e**) surface and (**f**–**j**) its cross-section. The yellow frame indicates the mapped portion.

**Figure 9:**
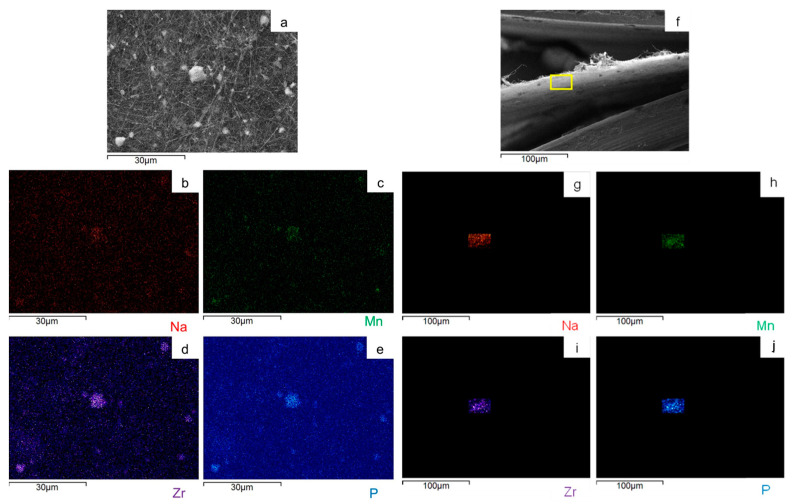
SEM image and EDS maps of the different elements for the v-30%MnZr/CNF (**a**–**e**) surface and (**f**–**j**) its cross-section. The yellow frame indicates the mapped portion.

**Figure 10:**
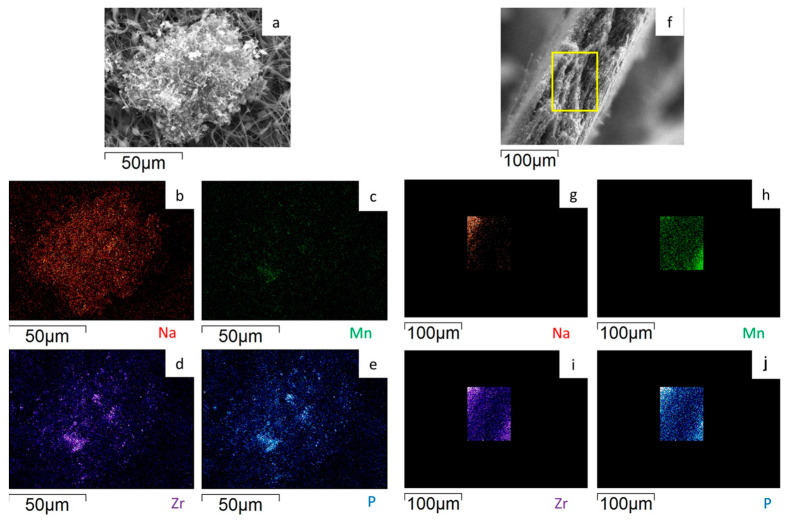
SEM image and EDS maps of the different elements for the dd-MnZr/CNF (**a**–**e**) surface and (**f**–**j**) its cross-section. The yellow frame indicates the mapped portion.

To evaluate the Na_3_MnZr(PO_4_)_3_ distribution on the surface and within the cross-section of the MnZr/CNF self-standing electrodes, an EDS analysis was performed. For each sample, a surface and a cross-section portion were selected, and the distribution maps of the Na, Mn, Zr, and P elements were evaluated. The results are shown in Figure 7, Figure 8, Figure 9 and Figure 10 for the h-10%MnZr/CNF, h-30%MnZr/CNF, v-30%MnZr/CNF, and dd-MnZr/CNF self-standing cathodes. In the case of the active material loaded by dispersion into the polymeric solution, the EDS analysis demonstrated that the agglomerates dispersed into and within the CNFs corresponded to Na_3_MnZr(PO_4_)_3_ composition. They were homogeneously distributed on the sample surface and along the sheet thickness too (Figure 7, Figure 8 and Figure 9). For the dd-MnZr/CNF sample (Figure 10), again the Na_3_MnZr(PO_4_)_3_ agglomerates were detected on the surface and in the cross-section, but their distribution along the sheet thickness was non-homogeneous, and the active material preferentially resided on the external edges. This justified the clearly visible Raman fingerprint of Na_3_MnZr(PO_4_)_3_ in the dd-MnZr/CNF composite compared to the other self-standing cathodes and was consistent with the dip–drop synthetic approach used to load the active material: the permeation of the precursors’ solution into the inner part of the pre-synthesized CNF sheets was less effective. The different concentration of the active material along the sheet may have influenced the electrochemical performance of the self-standing electrode. The atomic percentage of the Na, Mn, and P elements and their ratio were evaluated by EDS elemental analysis: independently of the self-standing MnZr/CNF cathodes, the Na:Mn:P mole ratio was close to 3:1:3, as expected from the compound’s stoichiometry.

The thermogravimetric data were used to evaluate the effective amount of the Na_3_MnZr(PO_4_)_3_ active material present in the self-standing cathodes. The thermal behavior of the pristine Na_3_MnZr(PO_4_)_3_ powder was also investigated. 

**Figure 11:**
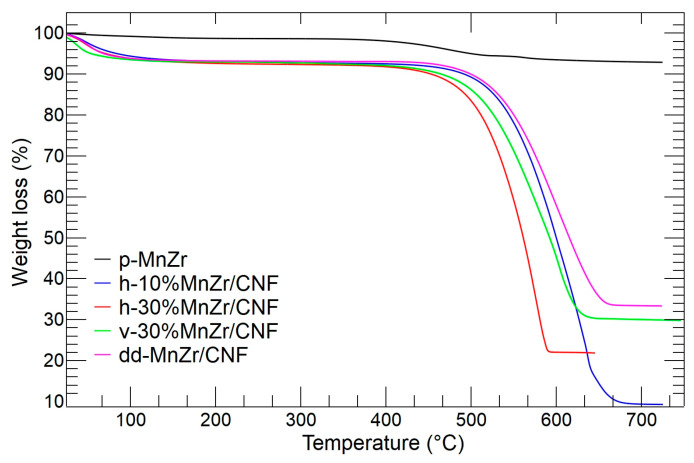
TGA analysis of p-MnZr (black), h-10%MnZr/CNF (blue), h-30%MnZr/CNF (red) v-30%MnZr/CNF (green), and dd-MnZr/CNF (purple) samples.

The thermogravimetric curves of the p-MnTi, h-10%MnZr/CNF, h-30%MnZr/CNF, v-30%MnZr/CNF, and dd-MnZr/CNF samples are shown in Figure 11. For the p-MnZr sample (black line), a small mass loss due to the release of adsorbed water was detected below 100 °C, while the second mass loss of 5.13% occurring in the temperature range 400–650 °C was attributed to the combustion of the carbon coating (source: citric acid used in sol–gel synthesis).

The h-10%MnZr/CNF (blue line), h-30%MnZr/CNF (red line), v-30%MnZr/CNF (green line), and dd-MnZr/CNF TGA curves displayed a similar thermal behavior. As for the p-MnZr sample, the first mass loss below 100 °C was attributed to the adsorbed water release. The second mass loss in the 400–650 °C temperature range was again due to the combustion of the carbonaceous component present as both the carbon coating and the carbon nanofibers. So, it was expected that this mass loss would be higher for MnZr/CNFs than the p-MnZr sample. The residual mass values reached by each self-standing cathode are reported in Table 2. In the case of the h-10%MnZr/CNF self-standing electrode, the obtained value well matched the synthesis one. On the contrary, for h-30%MnZr/CNF, the value was lower. This may depend on the horizontal experimental setting used for the deposition: we noted the partial settling of particles agglomerates in the syringe. To achieve the 30 wt% loading, the vertical experimental setting was used to prepare the self-standing cathode. Indeed, for the v-30%MnZr/CNF sample, the mass loading matched the synthesis value. The highest active material loading was obtained for the dd-MnZr/CNF sample by electrospinning the polymeric solution containing the Na_3_MnZr(PO_4_)_3_ precursors. 

From the values of the electrode residual masses for the v-30%MnZr/CNF and dd-MnZr/CNF samples we evaluated an active material loading of about 1.9 mg/cm^2^, which is consistent with the literature values of self-standing electrodes [56,63].

The characterization results put into evidence the analogies/differences of the self-standing cathodes prepared by different synthetic approaches and settings. In all cases, the active material was successfully loaded into the carbon nanofibers. The direct dispersion of pre-synthesized Na_3_MnZr(PO_4_)_3_ powder into the polymeric solution before electrospinning seems a promising and feasible route for loading the active material. It was homogeneously dispersed both into and within CNFs. The effective loading was quantitative when using the vertical setting, which prevented the possible settling of the powder before the needle was reached. The dip–drop coating of the active material precursor solution onto electrospun carbon nanofibers allowed the load of the highest Na_3_MnZr(PO_4_)_3_ amount, but the product mainly resided on the CNF sheet surface and did not distribute homogeneously along the sheet thickness.

### 2.2. p-MnZr and MnZr/CNF Electrochemical Characterization

The cyclic voltammetry of the Na_3_MnZr(PO_4_)_3_ material presented two redox peaks at 3.6 V and 4.1 V related to the Mn^2+^/Mn^3+^ and Mn^3+^/Mn^4+^ redox couples, respectively [38]. They indicated the reversible extraction/insertion of two Na^+^ ions involving the two-phase redox processes reported in Equations (1) and (2) [38,41]:

(1) $$Na_{3}{Mn}^{2+}Zr{\left(PO_{4}\right)}_{3}\overset{3.6\;V}{↔}Na_{2}M{n^{3}}^{+}Zr{\left(PO_{4}\right)}_{3}+Na^{+}+e^{-}$$

(2) $$Na_{2}{Mn}^{3+}Zr{\left(PO_{4}\right)}_{3}\overset{4.1\;V}{↔}NaM{n^{4}}^{+}Zr{\left(PO_{4}\right)}_{3}+Na^{+}+e^{-}.$$

The presence of Zr^4+^ was only necessary to unlock the rare Mn^3+^/Mn^4+^ redox reaction useful to produce a high-voltage cathode for sodium-ion batteries [64]. 

Gao and coworkers [38] reported a deep investigation of the desodiation process based on ex situ X-ray diffraction structural analysis and density functional theory calculations. In the aforementioned process, the Na^+^ extraction mainly involves MnO_6_-Na(2)-ZrO_6_ sites leaving MnO_6_-Na(2)-MnO_6_ and MnO_6_-Na(1)-MnO_6_ occupied. During desodiation, the ZrO_6_-Na(1)-ZrO_6_ occupation increases and the Na(2) vacancies promote Na(1) displacement to the Na(3) site. The latter process leads to completely occupied Na(1) sites and unoccupied Na(2) ones.

Figure S3 reports the cyclic voltammetry and charge/discharge curves for the p-MnZr slurry and MnZr/CNF self-standing electrodes. The p-MnZr slurry electrode data (Figure S3a,b) well compared to the literature ones [38]: the two redox peaks of Mn^2+^/Mn^3+^ and Mn^3+^/Mn^4+^ were detected at 3.61 V/3.45 V and 4.19 V/4.12 V, respectively. The small ΔV values between the cathodic and anodic peaks (16 mV for Mn^2+^/Mn^3+^ and 7 mV for Mn^3+^/Mn^4+^) indicated a very small polarization phenomenon. The current intensity was higher than 0.05 A/g and lower than −0.03 A/g for the anodic and cathodic peaks, respectively. The two redox phenomena were also confirmed by the two plateaus detected at 4.2 V and 3.5 V in the charge/discharge curves. 

In the case of the self-standing cathodes, it was difficult to individuate the manganese redox peaks, as they were very faint. This may have depended on the active material amount, which was lower than that used in the tape-cast cathode. Only in the v-30%MnZr/CNF sample, the weak cathodic peaks at 3.44 V and 4.05 V were detected and confirmed by the presence of the small plateau of Mn^3+^/Mn^4+^ at about 4.1 V in the charge/discharge curves (Figure S3g,h). We recall that, among the cathodes prepared by the dispersion of pre-synthesized Na_3_MnZr(PO_4_)_3_, the v-30%MnZr/CNF sample had the highest active material loading (29.8 wt%). For the dd-MnZr/CNF cathode, a comparable Na_3_MnZr(PO_4_)_3_ amount (33.0 wt%) was detected, but the active material was not embedded into the CNFs and non-homogeneously dispersed along the CNFs’ thickness, as demonstrated by the TEM and EDS analyses: this may have affected the electrochemical performance.

**Figure 12:**
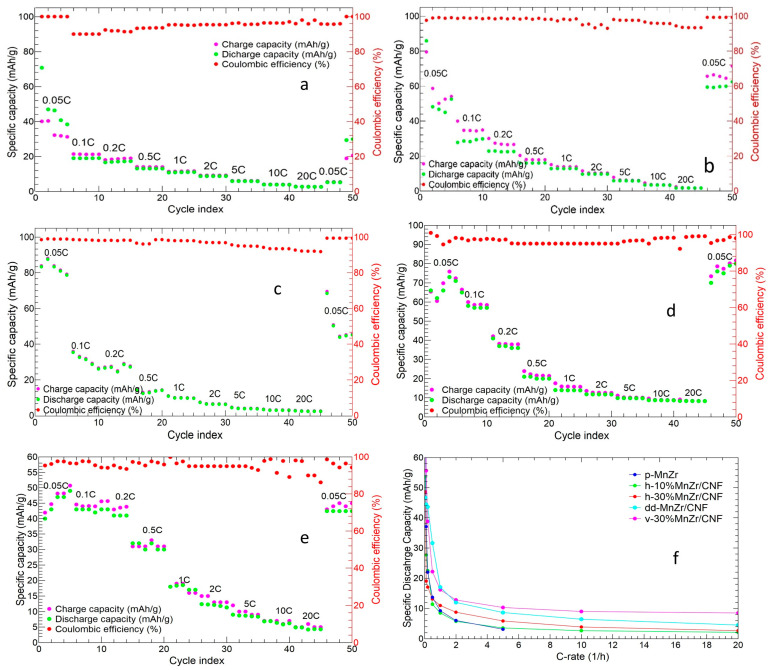
Charge/discharge cycles at different C-rates of (**a**) p-MnZr, (**b**) h-10%MnZr/CNF, (**c**) h-30%MnZr/CNF, (**d**) v-30%MnZr/CNF, and (**e**) dd-MnZr/CNF; (**f**) comparison of average discharge capacity values for all the samples at different C-rates.

Figure 12 shows the charge/discharge cycles at different C-rates for the slurry and for the MnZr/CNF self-standing cathodes. The tape-cast electrode showed an initial charge and discharge capacity of 105.88 and 67.21 mAh/g, respectively (Figure 12a). The initial capacity loss was typical of the NASICON-structured cathodes containing Zr^4+^ or Ti^4+^ or Al^3+^, which presented a voltage hysteresis of Al^3+^ < Ti^4+^ < Zr^4+^ [64]. Average discharge capacities of 58.62, 34.61, 21.19, 13.27, 9.01, 5.87, and 3.01 Ah/g at 0.05C, 0.1C, 0.2C, 0.5C, 1C, 2C, and 5C were obtained, respectively. The cell did not completely recover the capacity at the end of the measurement (a capacity of 24.18 mAh/g was reached at 0.05C), and the Coulombic efficiency (CE%) was ≥93%.

The h-10%MnZr/CNF cathode exhibited an initial charge and discharge capacity of 40.1 and 70.7 mAh/g, respectively (Figure 12b), with a discharge capacity which reached a value of 46.9 mAh/g in the second cycle. We obtained average discharge capacities of 48.6, 19.0, 17.0, 13.1, 11.0, 8.8, 5.9, 3.9, and 2.7 mAh/g at 0.05C, 0.1C, 0.2C, 0.5C, 1C, 2C, 5C, 10C, and 20C, respectively, and a Coulombic efficiency ≥95%. Contrary to the slurry electrode, the self-standing h-10%MnZr/CNF cathode first exhibited a lower loss capacity as the C-rate increased and, secondly, worked at C-rates higher than 5C and completely recovered the initial capacity at the end of the measurement. Indeed, the 10% self-standing cathode displayed capacity values at 10C comparable to those obtained for the p-MnZr slurry at 5C and a better Coulombic efficiency, which confirmed a better reversibility of the charge and discharge process.

Improved electrochemical performance was obtained for the h-30%MnZr/CNF sample (Figure 12c), which displayed an initial charge and discharge capacity of 83.9 mAh/g and 83.4 mAh/g, respectively. In this case, the initial capacity loss after the first cycle was not detected, and a Coulombic efficiency ≥97% was reached, but the capacity was not completely recovered at the end of the measurement (capacity of 45.2 mAh/g). The average discharge capacities of 53.6, 27.6, 22.5, 11.4, 8.5, 5.8, 3.6, 2.7, and 2.1 mAh/g were obtained at 0.05C, 0.1C, 0.2C, 0.5C, 1C, 2C, 5C, 10C, and 20C, respectively. These discharge capacity values were higher than the ones reported for the h-10%MnZr/CNF sample at 0.05C, 0.1C, and 0.2C. This was probably due to the higher amount of p-MnZr powder loaded into the CNF sheets (21.8 wt%). 

Figure 12d shows the charge and discharge cycles at different C-rate for the v-30%MnZr/CNF sample. It exhibited an initial charge and discharge capacity of 65.4 mAh/g and 100.9 mAh/g, respectively, and the discharge value approached the theoretical one (107 mAh/g) [38]. As for h-30%MnZr/CNF, no initial discharge capacity loss was detected, and the sample completely recovered the initial capacity at the end of the measurement with a Coulombic efficiency ≥96%. The average values of discharge capacity at 0.05C, 0.1C, 0.2C, 0.5C, 1C, 2C, 5C, 10C, and 20C were 78.2, 55.7, 38.8, 22.2, 16.2, 12.8, 10.3, 9.0, and 8.5 mAh/g, respectively. These values were higher at each C-rate than those for the h-10%MnZr/CNF and h-30%MnZr/CNF samples thanks to the higher amount of p-MnZr powder loaded into the CNF sheets.

Finally, the charge/discharge cycles at different C-rates of the dd-MnZr/CNF sample are shown in Figure 12e. The initial charge and discharge capacities were 41.9 and 40.0mAh/g, respectively, similar to the value of the h-10%MnZr/CNF cathode. In this case, the capacity was rather stable at low C-rates, and the capacity loss became remarkable only at C-rates higher than 0.2C. Indeed, the average capacity values were 46.7, 44.5, and 43.6 mAh/g at 0.05C, 0.1C, and 0.2C, 31.6 and 17.0 mAh/g at 0.5C and 1C, and 12.0, 8.7, 6.5, and 4.5 mAh/g at 2C, 5C, 10C, and 20C, respectively. The capacity was completely recovered at the end of the measurement, and a Coulombic efficiency ≥94% was reached. Hence, the dd-MnZr/CNF self-standing cathode displayed better electrochemical performances than the h-MnZr/CNF electrodes but worse than the v-30%MnZr/CNF one. These results can be explained by the higher amount of active material (33 wt%) present in the CNFs compared to that in the horizontal samples (9.3 and 21.8 wt%). However, the active materials aggregates were non-homogeneously distributed along the CNF sheets’ thickness and were preferentially located on their surface, and this undoubtedly affected the electrochemical performance. The charge/discharge investigation suggested a superior performance of all the self-standing cathodes in terms of a specific capacity at high C-rates and Coulombic efficiency compared to the tape-cast counterpart. The Coulombic efficiency values of all the MnZr/CNFs electrodes approached the CE% reported in the literature for other binder-free electrodes tested for cycles at different C-rates, typically ranging between 95% and 99% [55,65,66]. Notably, the v-30%MnZr/CNF cathode exhibited both high loading (29.8 wt%) and homogeneous distribution of the active material, and improved electrochemical performances were expected.

Figure 12f compares the average discharge capacities of all the investigated cathodes at the different C-rates. All the MnZr/CNF self-standing electrodes exhibited improved electrochemical performances compared to the p-MnZr slurry cathode. Indeed, independently of the Na_3_MnZr(PO_4_)_3_ amount in the CNF sheets, the self-standing electrodes were performant at C-rates higher than 5C, and the higher the active material amount, the higher the capacity value at each C-rate. These results confirmed the beneficial role of CNFs, especially at high C-rates, when the required fast ion diffusion is invalidated in packed materials such as tape-cast cathodes [57,58]. However, among the three approaches applied to synthesize the self-standing electrodes, the dip–drop method gave intermediate values between the horizontal and vertical settings in terms of both the capacity value and the electrochemical performance at a high C-rate. The best electrochemical results were obtained for the v-30%MnZr/CNF cathode, which exhibited not only the highest initial capacity values but also the best performance at high C-rates. The reported results confirmed that the vertical set-up provided the best self-standing electrode. Based on this, we completed our investigation by testing the long cycling capability and lifespan of the v-30%MnZr/CNF sample compared to the slurry one. We wanted to confirm that the right synthesis of a self-standing electrode gives a cathode with longer cycling and better performances at high C-rates than the slurry one, thanks to CNFs’ properties. 

**Figure 13:**
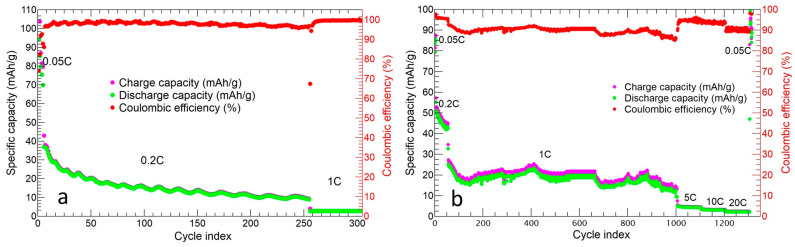
Cycling performance of (**a**) p-MnZr slurry electrode and (**b**) v-30%MnZr/CNF self-standing cathode.

The long charge/discharge cycling of the two cathodes is shown in Figure 13.

The tape-cast cathode (Figure 13a) was tested at 0.05C, 0.2C, and 1C for 5, 200, and 100 cycles. After the initial charge and discharge specific capacity of 104.3 mAh/g and 94.2 mAh/g, respectively, the cell underwent a specific capacity loss: a value of 16.5 mAh/g was achieved during the 82nd cycle at 0.2C. This specific capacity value was quite constant up until the 200th cycle at 0.2C, with a capacity retention of 15.8% and an average Coulombic efficiency ≥98%. When the cell was tested at 1C, the specific capacity decreased down to 2.6 mAh/g and could not be further cycled at 0.05C.

The v-30%MnZr/CNF self-standing cathode was tested at 0.05C, 0.2C, 1C, 5C, 10C, and 20C for 5, 50, 1000, 100, 50, and 30 cycles, respectively (Figure 13b). The initial charge and discharge capacities at 0.05C were 87.2 mAh/g and 85.4 mAh/g, respectively. Then, a discharge capacity loss was detected (45.2 mAh/g at 0.2C for 50 cycles), as well as average values of the discharge capacity of 19.3 mAh/g at 1C, 4.2 mAh/g at 5C, 3.7 mAh/g at 10C, and 2.5 mAh/g at 20C. The average Coulombic efficiency was ≥91%, approaching those obtained for other electrode materials, such as NaVPO_4_F/CNF after 50 cycles [53]. The capacity was completely recovered at the end of the measurement in the last cycle at 0.05C, and the capacity retention at 0.2C for 50 cycles and at 1C for 1000 cycles was 52.9% and 22.6%, respectively. We can conclude that, contrary to the p-MnZr slurry cathode, the v-30%MnZr/CNF self-standing electrode presented a better capacity value at each C-rate, and it worked at a high C-rate after 1000 cycles at 1C too. Moreover, it also showed better capacity retention at both 0.2C and 1C. 

An electrochemical impedance spectroscopy (EIS) analysis was performed on the v-30%MnZr/CNF and p-MnZr cathodes to further investigate the effect of CNFs on the kinetic process of the self-standing electrodes. The Nyquist plot and the equivalent circuit are shown in Figure S4 (Supplementary Materials). The EIS spectra were consistent with those reported in the literature for the Na_3_MnZr(PO_4_)_3_ active material [41]. The smaller diameter of the semicircle in the high-frequency region for the v-30%MnZr/CNF electrode suggested a smaller charge transfer resistance (807 Ω vs. 1092 Ω of the tape-cast cathode) and a faster charge transfer at the electrode–electrolyte interface. Moreover, the decrease in electrolyte resistance in the self-standing electrode (10.7 Ω vs. 24.2 Ω for the tape-cast counterpart) suggested a better electrolyte permeation into MnZr/CNF electrodes, thanks to the presence of a CNF porous matrix. Finally, the larger slope of Warburg impedance for v-30%MnZr/CNF than for the p-MnZr cathode indicated that the more favorable Na ion transport was in the self-standing electrode.

The reported results put into evidence the advantages of using CNFs to assemble cathodes for SIBs. An improved electrochemical performance was obtained, as they enhanced the electronic conductivity and guaranteed good electrolyte–active material contact, thanks to the easy permeation of the electrolyte into their CNF porous matrix. Notably, the CNFs demonstrated to be a suitable support to realize free-standing electrodes, allowing us to avoid the use of Al foils as the current collectors. Among the synthetic approaches to prepare electrospun active material–carbon nanofibers cathodes (see Figure 1), better electrochemical performances in terms of capacity values and lifespan were obtained for a higher Na_3_MnZr(PO_4_)_3_ amount, reached by using a vertical setting in the electrospinning process: in this case, the active material particles, pre-synthesized via sol–gel, were quantitatively deposited in the CNF sheets, compared to the horizontal setting. When a comparable active material amount was loaded into CNFs (v-30%MnZr/CNF and dd-MnZr/CNF samples), the best performances were obtained by dispersing the pre-synthesized Na_3_MnZr(PO_4_)_3_ powder into the polymeric solution to be electrospun, as this approach allowed a homogeneous dispersion of the active material along the CNF sheets, and the agglomerates were present both within and embedded into the CNFs. On the contrary, in the dip–drop approach, the active material was preferentially distributed on the sheets’ surface and on the external surface of the carbon nanotubes.

## 3. Materials and Methods

### 3.1. Materials

The chemicals (Signa-Aldrich, Milano, Italy) were of a reagent-grade or higher quality. Citric acid (C_6_H_8_O_7_), ammonium phosphate monobasic (NH_4_H_2_PO_4_), sodium acetate (CH_3_COONa) manganese(II) acetate tetrahydrate ((CH_3_COO)_2_Mn · 4H_2_O), zirconium(IV) acetylacetonate (Zr(C_5_H_7_O_2_)_4_), 1M sodium perchlorate in PC (1:1 *v*:*v*) electrolyte, fluoroethylene carbonate (FEC), carbon acetylene black powder, poly(vinylidene fluoride) (PVdF), polyacrylonitrile (PAN: (C_3_H_3_N)_n_), and N,N-dimethylacetamide (DMAc: CH_3_CON(CH_3_)_2_) were employed.

### 3.2. Synthesis

#### 3.2.1. Active Material Na_3_MnZr(PO_4_)_3_

The Na_3_MnZr(PO_4_)_3_ synthesis procedure reported in [38] was followed. Based on Na_3_MnZr(PO_4_)_3_ stoichiometry, an aqueous solution of CH_3_OONa, (CH_3_COO)_2_Mn · 4H_2_O, NH_4_H_2_PO_4_, and Zr(C_5_H_7_O_2_)_4_ was prepared. To the solution was added citric acid (citric acid moles equals the total transition metal ones) as the chelating agent, in order to obtain the carbon coating of the active material aggregates. The solution was heated at 80 °C to obtain the gel that was dried at 100 °C in an oven. The product was ground in an agate mortar and treated at 750 °C for 10h under a nitrogen flux in a tube furnace (Carbolite). The final powder product (pristine product) was called p-MnZr.

#### 3.2.2. Self-Standing Cathodes

As reported in Section 2 and Figure 1, two different approaches were proposed to prepare the self-standing electrodes, differing in the method employed to load the active material onto CNFs and the electrospinning setting. For the horizontal depositions, a EF050-Starter Kit Electrospinning of SKE Research Equipment (C/O Leonardino S.r.l, Bollate, MI, Italy) was used. A home-made humidity control box was used to achieve humidity values lower than 20%. An electrospinning apparatus, NANON01A, equipped with a dehumidifier (MEEC instruments, MP, Pioltello, Italy) was employed to produce vertical depositions.

In the dip–drop procedure, the pure carbon nanofibers (CNF sample) were prepared first: 8 wt% PAN solution in DMAc was electrospun (horizontal setting) with 3.5 mL/h rate, 16 gauge needle, applied voltage 14 kV, and a needle–collector distance of 18cm. Once the collected CNF sheets were removed from the support (aluminum foil), the CNFs were thermally treated: for the stabilization process, the CNFs were heated for 30 min at 100 °C, for 30 min at 200 °C, and, finally, for 2 h at 260 °C (heating ramp: 5 °C min^−1^) in air, while the carbonization process was conducted in a tubular furnace (Carbolite) at 900 °C for 2 h (heating ramp: 10 °C min^−1^) under a nitrogen atmosphere. For the dip–drop process, a solution containing the active material precursors and citric acid was prepared following the reagent quantities used in sol–gel synthesis (see Section 3.2.1). The carbonized CNF sheet was deeply drenched in the solution of active material reagents, then the same solution was dropped on the CNF sheet surface. The Na_3_MnZr(PO_4_)_3_ precursor-soaked-CNF sheet was dried under a vacuum at 120 °C for one night and thermal-treated like the sol–gel sample: 750 °C for 10 h under a nitrogen atmosphere. The obtained self-standing cathode was called dd-MnZr/CNF.

In the second synthetic approach, the Na_3_MnZr(PO_4_)_3_ powder (p-MnZr sample) was ball-milled at 100 rpm for two cycles (20 min each), then 10 wt% (0.188g) or 30 wt% (0.564g) of p-MnZr was added to 25 mL of N, N-dimethylacetamide, and the suspension was sonicated 1 h. Then, 1.88 g PAN was added, and the suspension was stirred overnight at 60 °C. The obtained dispersions were electrospun using a horizontal setting apparatus (samples called h-10%MnZr/CNF and h-30%MnZr/CNF). The 30 wt% dispersion was also electrospun employing a vertical equipment (sample named v-30%MnZr/CNF). Using horizontal deposition electrospinning, the setting parameters were optimized as follows: 10.5 mL dispersion, 3.5 mL/h flow, 16 gauge needle, applied voltage 18 kV, needle–collector distance 18 cm, deposition time 3 h, temperature and relative humidity values equal to 25 °C and lower than 20%, respectively, during each deposition. Instead, for vertical deposition electrospinning, the parameters were set as follows: 10 mL dispersion, 0.5 mL/h flow rate, 16 gauge needle, applied voltage 28 kV, needle–collector distance 15 cm, deposition time 3h, and temperature and relative humidity values equal to 23 ± 2 °C and 20 ± 3%, respectively, during each deposition.

Both horizontally and vertically deposited self-standing electrodes were removed from the support and underwent stabilization (30 min at 100 °C, 30 min at 200 °C, and 2 h at 260 °C in air) and the carbonization process at 750 °C in N_2_ flux. 

#### 3.2.3. Tape-Cast Cathode

The p-MnZr active material was first ball-milled at 100 rpm for two cycles (20 min each). The slurry was obtained by dispersing 70 wt% active material, 20 wt% acetylene carbon, and 10 wt% PVdF binder (Solvay 5130) in distilled water. The slurry was magnetically stirred for 2 h, and then it was tape-cast (Doctor Blade coating technique) on aluminum foils and dried at 70 °C for 3 h. 

### 3.3. Cell Assembly

Swagelok-type cells were assembled in an argon-filled dry box (MBraun; H_2_O content < 0.1 ppm, O_2_ content < 0.1 ppm). NaClO_4_ 1M in PC and 5 wt% FEC was used as the electrolyte, and sodium foil was used as the counter-electrode. The working and counter-electrode showed a diameter of 1cm.

### 3.4. Characterization Techniques

For the X-ray powder diffraction (XRPD) analysis, a Bruker D5005 diffractometer (Bruker, Karlsruhe, Germany) equipped with a Cu Kα radiation tube (40 kV, 40 mA), a curved graphite monochromator on the diffracted beam, and a scintillation detector was used. The patterns collection conditions were the following: 16–80° 2-theta range, 0.03° step size, and 22 s/step counting time. The TOPAS 3.0 software [67] was used for the Rietveld structural refinement of the p-MnZr and MnZr/CNFs samples.

Room temperature micro-Raman measurements were performed using an integrated confocal micro-Raman spectrometer, XploRA Plus HORIBA Scientific, equipped with an Olympus microscope BX43. Green laser light at 532 nm was used for excitation tuning the 100 mW output power through a set of neutral filters with different optical densities. The samples were positioned on a motorized xy stage automatically controlled, allowing mapping acquisitions. The spectral resolution was about 3 cm^−1^. The measurements were performed using a long work distance 50× magnification objective with a spatial resolution of the order of 4 microns. The spectra were acquired with a mean integration time of about 10 s and a number of accumulations equal to 10. All the reported data were obtained as an average spectrum, sampling the materials in several different regions.

The scanning electron microscopy images were taken with a Zeiss EVO MA10 (Carl Zeiss, Oberkochen, Germany) microscope, equipped with an energy dispersive detector for EDS analysis, on gold-sputtered samples (20 kV, secondary electrons images, working distance of 8.5 mm). 

The transmission electron microscopy images were collected with a JEOL JEM-1200EXIII equipped with a TEM CCD camera Mega View III transmission electron microscope. The samples were dispersed in water; a drop of about 0.7 μL was deposited on the formvar/carbon-coated Ni grid and dried. 

For the thermogravimetric analysis, a TGA Q5000 IR apparatus interfaced with a TA 5000 data station (TA Instruments, Newcastle, DE, USA) was employed. The thermogravimetric data were collected in the 25–750 °C temperature range in air (heating rate: 10 °C min^−1^). 

The electrochemical properties of the tape-cast and self-standing cathodes were investigated at room temperature on a Swagelok cell. For cyclic voltammetry (CV), an Autolab PGSTAT30 potentiostat (Eco Chemie) in the 2.5–4.5 V potential range was used, and the data were processed with the GPES V4.9 software. For the galvanostatic charge/discharge cycles, we used a Neware-4000BTS battery testing system at different current rates in the 2.5–4.5 V potential range. The electrochemical impedance spectroscopy (EIS) measurements were performed on an Autolab PGSTAT30 potentiostat (Eco Chemie). The EIS spectra were acquired at OCV in the 10^6^–10^−2^ Hz frequency range with an amplitude potential of 1mV.

## 4. Conclusions

In this study, different synthetic approaches based on the electrospinning technique were applied to prepare Na_3_MnZr(PO_4_)_3_/CNF free-standing cathodes for SIBs. Independently of the method employed, the active material was successfully loaded into CNFs and its NASICON-type crystal structure was maintained. Indeed, the synthesis route had an impact on the loaded active material amount and distribution through the carbon nanofiber sheets. Compared to the dip–drop coating, the dispersion of the pre-synthesized active material method allowed the homogeneous distribution of the Na_3_MnZr(PO_4_)_3_ particles along the thickness of the sheet, and the aggregates connected and covered the surface of the CNFs but were also embedded into them: this active material–CNF intimate contact resulted beneficial to the electrochemical performances. Concerning the setting, the vertical one seemed more efficient, as particulate decantation was avoided, and the active material was quantitatively loaded into the CNFs. Indeed, the v-30%MnZr/CNF sample displayed the best electrochemical performances. 

Independently of the synthetic approach, an improved electrochemical performance in terms of specific discharge capacities at different C-rates was achieved in the self-standing cathodes compared to the tape-cast one. Notably, the enhancement was particularly evident at high C-rates, where the porous nature of the non-woven nanofibers guaranteed electrolyte diffusion and easy contact with the active material aggregates. The best electrochemical performances were achieved by the v-30%MnZr/CNF electrode, which also demonstrated a promising long cycling life compared to the tape-cast counterpart. The reported results suggest that the ex situ synthesis of Na_3_MnZr(PO_4_)_3_ and its addition (30 wt%) to the carbon precursor solution to be electrospun with a vertical setting are a simple and feasible approach to obtaining self-standing cathodes with enhanced electrochemical performances compared to tape-casted cathodes (70 wt% of active material). 

## Figures and Tables

**Table 1:** List and preparation details of the investigated samples.

CODE	SAMPLE	DETAILS
CNF	Pure carbon nanofibers	Electrospinning Setting: horizontal
p-MnZr	Pristine Na_3_MnZr(PO_4_)_3_ powder	Sol–gel route
dd-MnZr/CNF	Self-standing cathode 33 wt% active material (from TGA)	Electrospinning Dip–drop method Setting: horizontal
h-10%MnZr/CNF	Self-standing cathode 10 wt% active material (from synthesis)	Electrospinning Active material dispersion Setting: horizontal
h-30%MnZr/CNF	Self-standing cathode 30 wt% active material (from synthesis)	Electrospinning Active material dispersion Setting: horizontal
v-30%MnZr/CNF	Self-standing cathode 30 wt% active material (from synthesis)	Electrospinning Active material dispersion Setting: vertical

**Table 2:** Residual mass values of the self-standing cathodes evaluated by TGA analysis.

SAMPLE	RESIDUAL MASS (wt%)
h-10%MnZr/CNF	9.3
h-30%MnZr/CNF	21.8
v-30%MnZr/CNF	29.8
dd-MnZr/CNF	33.3

## Data Availability

The data presented in this study are available upon request from the corresponding author.

## Supplementary Materials

The following supporting information can be downloaded at https://www.mdpi.com/article/10.3390/molecules29081885/s1: Figure S1: Rietveld refinement of the X-ray diffraction data of the p-MnZr sample; Figure S2: Rietveld refinement of the X-ray diffraction data of the MnZr/CNF samples; Figure S3: CV and charge/discharge profiles of the tape-cast and self-standing MnZr/CNF cathodes; Figure S4: Nyquist plot of the v-30%MnZr/CNF and p-MnZr cathodes; Table S1: Lattice parameters, cell volume, *c*/*a* ratio, crystallite size, and discrepancy factors obtained by the Rietveld refinement of the diffraction data of the pristine and CNF-loaded Na_3_MnZr(PO_4_)_3_ samples.
