# Supplementary material for: Design of Na3MnZr(PO4)3/Carbon Nanofiber Free-Standing Cathodes for Sodium-Ion Batteries with Enhanced Electrochemical Performances through Different Electrospinning Approaches
Source: Molecules. 2024 Apr 20;29(8):1885. doi: 10.3390/molecules29081885 (PMC11053439; doi:10.3390/molecules29081885)
Supplement: Supplementary file 1 [file molecules-29-01885-s001.zip › molecules-2946456-supplementary.pdf]

Supplementary

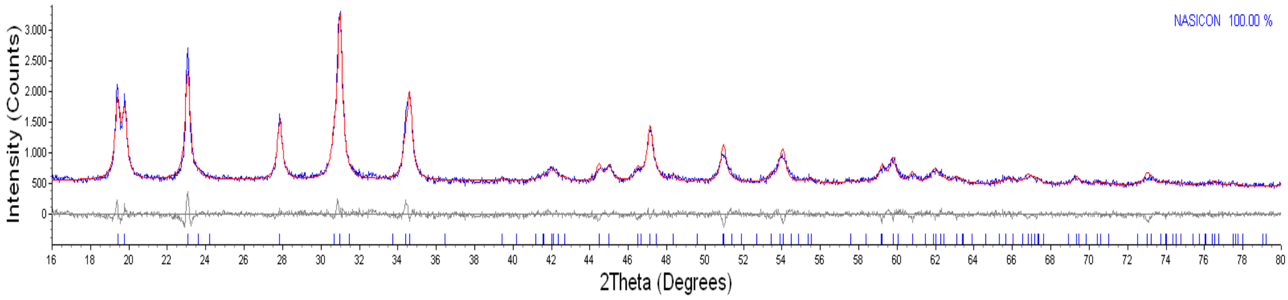

Figure S1. Rietveld refinement of the X-ray diffraction data of the p-MnZr sample. Experimental pattern (blue line), calculated pattern (red line), difference curve (grey line). Peaks position of the $Na_3MnTi(PO_4)_3$ phase (blue bars on the bottom).

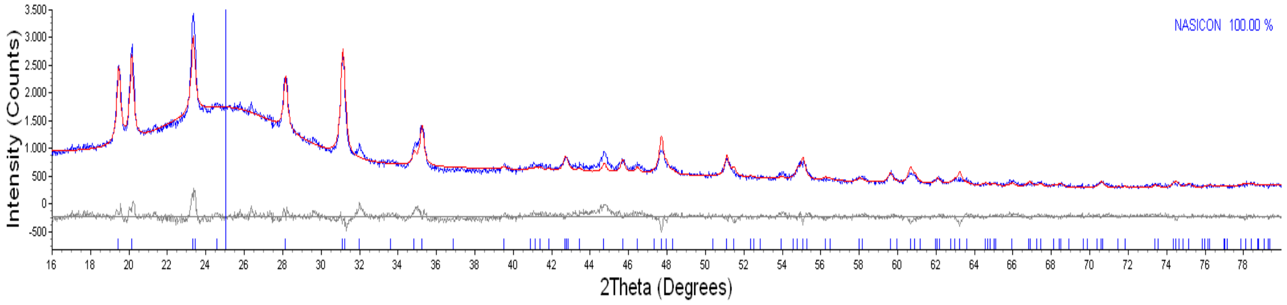

(a)

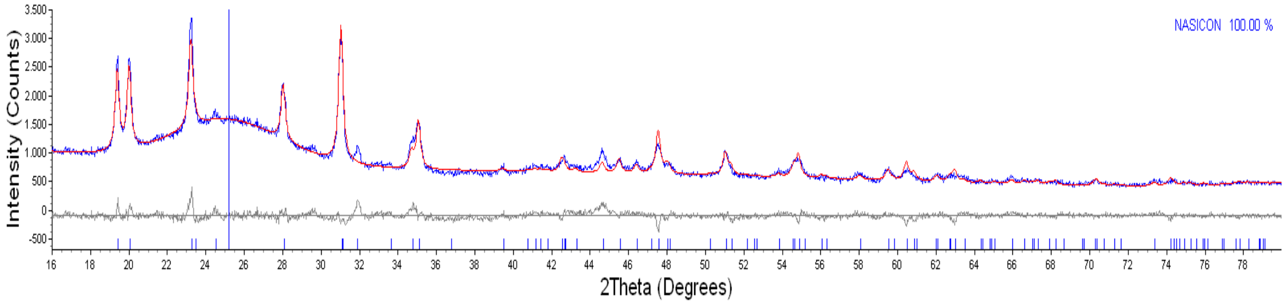

(b)

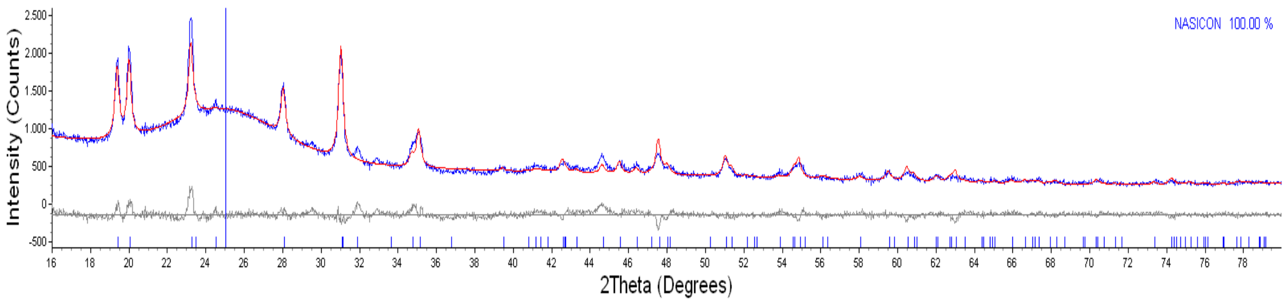

(c)

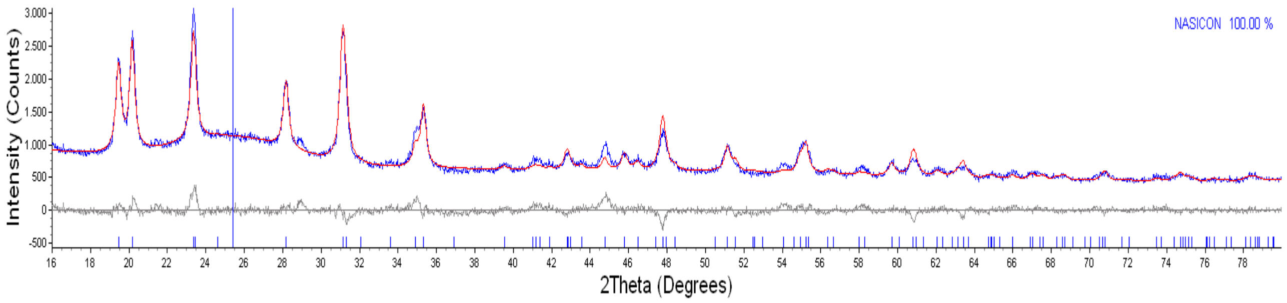

(d)

Figure S2. Rietveld refinement of the X-ray diffraction data of the a) h-10%MnZr/CNF, b) h-30%MnZr/CNF c) v-30%MnZr/CNF and d) dd-MnZr/CNF samples. Experimental pattern (blue line), calculated pattern (red line), difference curve (grey line). Peaks position of the $Na_3MnTi(PO_4)_3$ phase (blue bars on the bottom). The refined position of the broad peak of the amorphous CNF phase is indicated by the blue vertical line.

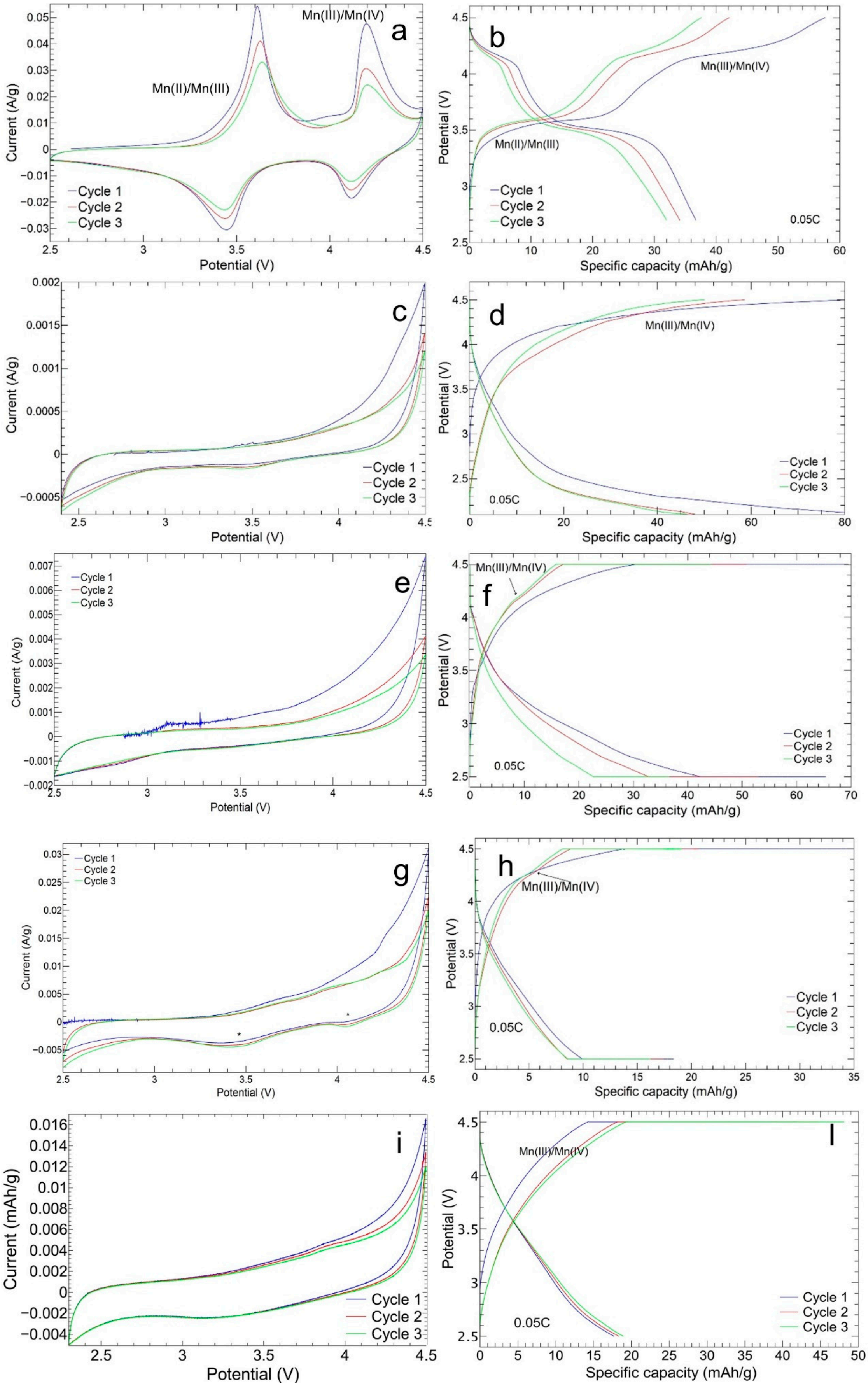

Figure S3. CV and charge/discharge curves of slurry p-MnZr (a,b), h-10%MnZr/CNF (c,d), h-30%MnZr/CNF (e,f), v-30%MnZr/CNF (g,h), and dd-MnZr\CNF (i,l) cathodes.

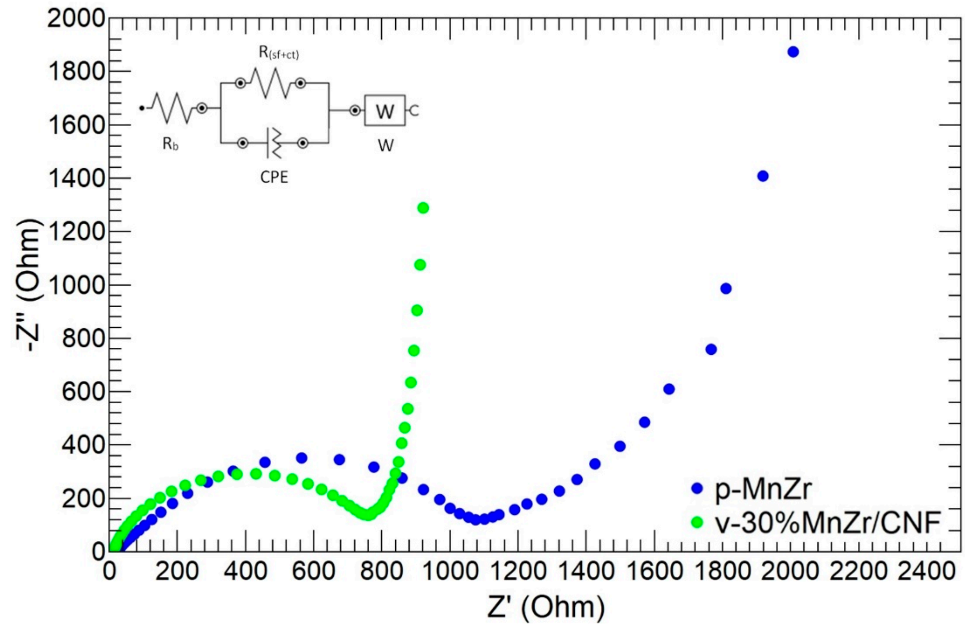

Figure S4. Nyquist plot of the v-30%MnZr/CNF and p-MnZr cathodes. The equivalent circuit is shown in the inset. R_b: electrolyte resistance; $R_{(sf+ct)}$: surface and charge transfer resistance; W: Warburg impedance.

Table S1. Refined lattice parameters, cell volume, crystallite size, weighted-pattern discrepancy factor and Goodness of Fit of the NASICON-structured $Na_3MnZr(PO_4)_3$ phase obtained by Rietveld refinement of MnTi and MnTi/CNF samples.

**SAMPLE**	**p-MnZr**	**dd-MnZr/CNF**	**h-10%MnZr/CNF**	**h-30%MnZr/CNF**	**v-30%MnZr/CNF**
a (Å)	8.970(1)	8.794(1)	8.818(1)	8.844(1)	8.840(1)
c (Å)	22.585 (5)	22.742(4)	22.740(4)	22.705(4)	22.709(4)
V (Å^3)	175.45	173.20	173.66	173.90	173.85
c/a	2.518	2.586	2.578	2.567	2.569
Crystallite size (nm)	30(1)	28(1)	40(1)	37(1)	36(1)
R_{wp}	5.51	6.26	6.67	6.13	6.79
S	1.41	1.75	1.83	1.76	1.62
